# Transcriptional Activity and Protein Levels of Horizontally Acquired Genes in Yeast Reveal Hallmarks of Adaptation to Fermentative Environments

**DOI:** 10.3389/fgene.2020.00293

**Published:** 2020-04-30

**Authors:** Joaquín Devia, Camila Bastías, Eduardo I. Kessi-Pérez, Carlos A. Villarroel, Matteo De Chiara, Francisco A. Cubillos, Gianni Liti, Claudio Martínez, Francisco Salinas

**Affiliations:** ^1^Centro de Estudios en Ciencia y Tecnología de los Alimentos (CECTA), Universidad de Santiago de Chile (USACH), Santiago, Chile; ^2^Millennium Institute for Integrative Biology (iBio), Santiago, Chile; ^3^Departamento de Biología, Facultad de Química y Biología, Universidad de Santiago de Chile (USACH), Santiago, Chile; ^4^CNRS, INSERM, IRCAN, Université Côte d‘Azur, Nice, France; ^5^Departamento de Ciencia y Tecnología de los Alimentos, Facultad Tecnológica, Universidad de Santiago de Chile (USACH), Santiago, Chile; ^6^Instituto de Bioquímica y Microbiología, Facultad de Ciencias, Universidad Austral de Chile (UACH), Valdivia, Chile

**Keywords:** horizontal gene transfer, yeast, transcriptional activity, protein levels, fermentation

## Abstract

In the past decade, the sequencing of large cohorts of *Saccharomyces cerevisiae* strains has revealed a landscape of genomic regions acquired by Horizontal Gene Transfer (HGT). The genes acquired by HGT play important roles in yeast adaptation to the fermentation process, improving nitrogen and carbon source utilization. However, the functional characterization of these genes at the molecular level has been poorly attended. In this work, we carried out a systematic analysis of the promoter activity and protein level of 30 genes contained in three horizontally acquired regions commonly known as regions A, B, and C. In three strains (one for each region), we used the luciferase reporter gene and the *mCherry* fluorescent protein to quantify the transcriptional and translational activity of these genes, respectively. We assayed the strains generated in four different culture conditions; all showed low levels of transcriptional and translational activity across these environments. However, we observed an increase in protein levels under low nitrogen culture conditions, suggesting a possible role of the horizontally acquired genes in the adaptation to nitrogen-limited environments. Furthermore, since the strains carrying the luciferase reporter gene are null mutants for the horizontally acquired genes, we assayed growth parameters (latency time, growth rate, and efficiency) and the fermentation kinetics in this set of deletion strains. The results showed that single deletion of 20 horizontally acquired genes modified the growth parameters, whereas the deletion of five of them altered the maximal CO_2_ production rate (Vmax). Interestingly, we observed a correlation between growth parameters and Vmax for an ORF within region A, encoding an ortholog to a thiamine (vitamin B1) transporter whose deletion decreased the growth rate, growth efficiency, and CO_2_ production. Altogether, our results provided molecular and phenotypic evidence highlighting the importance of horizontally acquired genes in yeast adaptation to fermentative environments.

## Introduction

The widespread use of the budding yeast *Saccharomyces cerevisiae* as a eukaryotic model organism led to the sequencing of the genome of this microorganism more than 20 years ago (Goffeau et al., 1996). In addition, a broad range of biotechnological applications has encouraged the genome sequencing of numerous yeast strains associated with different productive activities (Borneman and Pretorius, 2015; Liti, 2015). Currently, the variety of sequenced strains includes isolates from domesticated settings, such as vineyards, breweries, cheese, dairy, and clinical environments as well as wild strains isolated from non-domesticated environments, such as forests, flowers, and tree bark (Liti et al., 2009; Novo et al., 2009; Borneman et al., 2011; Wang et al., 2012; Bergström et al., 2014; Strope et al., 2015; Legras et al., 2018; Peter et al., 2018). In this sense, the “1002 yeast genomes project” is the most comprehensive collection of the genetic variation of yeast, encompassing the genome information of 1011 isolates from diverse geographical origins and ecological niches and describing 26 clades within the yeast population structure (Peter et al., 2018).

The extensive amount of sequencing information accumulated so far has revealed a unique set of genetic features in the yeast genome, which are often related to their adaptation to specific niches (Legras et al., 2018). These genetic hallmarks are frequently absent in the yeast reference genome (lab strain S288C) and, in general, they have been acquired by introgression (probably through mating) from a closely related species or by Horizontal Gene Transfer (HGT) from a more distant species (Morales and Dujon, 2012). In the HGT process, the donor species has a distant phylogenetic relationship with the acceptor species, with both species coexisting in the same ecological niche (Fitzpatrick, 2012). In general terms, the HGT process in eukaryotes is triggered under specific (stressful) environmental conditions, where the acceptor species incorporates the foreign DNA into its genome by an unknown mechanism (Keeling and Palmer, 2008). Currently, detection of HGT is performed by phylogenetic methods to identify genes with a different evolutionary trajectory with respect to the host genes (Roelofs and Van Haastert, 2001; Keeling and Palmer, 2008; Ravenhall et al., 2015).

Multiple HGT events have been described in different yeast species, thus demonstrating the impact of these genomic regions on the adaptation to specific environments (Gojkovic et al., 2004; Novo et al., 2009; Kominek et al., 2019). After the sequencing of the *S. cerevisiae* genome was completed, at least 10 potential cases of HGT from bacteria were identified, including genes related to biotin utilization and pyrimidine biosynthesis (Gojkovic et al., 2004; Hall et al., 2005; Hall and Dietrich, 2007). Recently, the acquisition of an entire operon into the yeast clade *Wickerhamiella/Starmerella* from an ancient bacterium (close to the modern enterobacteriaceae) was described, allowing these yeasts to produce enterobactin and improving their adaptation to the insect gut environment (Kominek et al., 2019). Similarly, in the same clade, the thiamine (vitamin B1) biosynthesis pathway was assembled using genes and operons acquired by HGT from bacteria (Gonçalves and Gonçalves, 2019). This evidence has highlighted the importance of the transcriptional and translational activity of the genes acquired by HGT in the context of the acceptor species, in which the promoter structure, codon usage, and transcription and translation machineries differ at the molecular level between prokaryotes and eukaryotes. Furthermore, even if the two species (donor and acceptor) are eukaryotes, codon usage differences between species may impair the expression of the horizontally acquired genes.

Eukaryote-to-eukaryote HGT events have also been described in yeast, especially under the stressful conditions faced during the fermentation process. Analysis of the genome sequence of the French wine strain EC1118, widely used for Chardonnay wine production, revealed unique genetic features, including three regions not present in the reference genome: region A (38 Kb) carries 12 Open Reading Frames (ORFs), region B (17 Kb) contains 5 ORFs, and region C (65 Kb) harbors 17 ORFs (Novo et al., 2009). Region B was acquired from *Zygosaccharomyces bailii*, a species commonly found in grape must and that coexists with *S. cerevisiae* at the beginning of the fermentation process (Novo et al., 2009). Interestingly, region B replicates through a circular intermediate, explaining the copy number variations observed between yeast strains (Galeote et al., 2011). In a similar way, the subtelomeric regions A and C were acquired from the *Torulaspora* genus, and many species from this genus cohabit with *S. cerevisiae* at the beginning of grape fermentation (Marsit et al., 2015). Several functional genomics efforts have been performed to uncover the cellular and molecular processes in which these horizontally acquired genes play a role. For instance, region C contains the *FSY1* gene, which has been functionally characterized as a fructose transporter, showing the importance of this horizontally acquired gene in the fermentation process since fructose is one of the main sugars present in grape must (Galeote et al., 2010). The region C also includes ORFs related to oligopeptide transport (*FOT* genes), which confer an important adaptive advantage, expanding the sources of nitrogen utilized by wine yeasts during grape must fermentation (Marsit et al., 2015, 2016). Furthermore, region C contains the *XDH1* gene, which encodes for a xylitol dehydrogenase necessary for yeast growth in xylose (Wenger et al., 2010). However, beyond these examples, other genes within these regions have been putatively associated with nitrogen and carbon source transport and metabolism, processes in which they may play important roles in the adaptation of yeast to the fermentation process.

Horizontal gene transfer is not restricted to wine strains of *S. cerevisiae*, as recent sequencing projects have shown that HGT is an extended phenomenon present in yeast isolated from different ecological niches (Legras et al., 2018; Peter et al., 2018). The recent genome sequencing of 82 strains isolated from human-associated environments identified 42 horizontally acquired regions, which are under non-neutral evolution and are important for niche-specific adaptation (Legras et al., 2018). Similarly, several additional horizontally acquired regions were identified by the “1002 yeast genomes project.” Here, most of the isolates carry at least one candidate HGT region in the genome (Peter et al., 2018). Current sequencing information has become a powerful resource for functional genomics, allowing researchers to explore the molecular roles of horizontally acquired genes. However, an important aspect is the molecular detection of the functional activation of these genes, which is of critical importance if the horizontally acquired genes are to participate in yeast adaptation to different environments. So far, there has been a scarce amount of molecular evidence supporting this assumption.

In this work, we used information from the “1002 yeast genomes project” to select three strains carrying the horizontally acquired regions A, B, or C. In these strains, we systematically assayed the transcriptional and translational activity of all the HGT genes, using both the luciferase reporter gene and the *mCherry* fluorescent protein, respectively. These two collections of transformed strains were assayed in four different culture conditions, and while they showed low levels of transcriptional and translational activity across the environments, we observed a general increase in protein levels under nitrogen-limited conditions. Furthermore, we showed that single deletion of several horizontally acquired genes impairs the growth parameters (latency time, growth rate, and efficiency) and fermentation kinetics. Overall, our results provided molecular and phenotypic evidence confirming the substantial role of the horizontally acquired genes in yeast adaptation to the fermentative environment.

## Materials and Methods

### Strains and Culture Conditions

The three strains used in this work (ALL, AHG, and BSS) were part of the “1002 yeast genomes project” (Peter et al., 2018). These strains belong to the Wine/European clade and were isolated from different geographical and ecological origins (Supplementary Table 1). These strains were selected based on the information of copy number for each horizontally acquired region (A, B, or C) per haploid genome described by Peter et al. (2018). Specifically, ALL carries a hemizygotic region A in its genome, AHG is haploid and contains region B, whilst BSS harbors region C in hemizygosis (Supplementary Table 2). All the strains used and generated in this work are listed in the Supplementary Table 1.

The strains were maintained in media YPDA (2% glucose, 2% peptone, 1% yeast extract, and 2% agar) at 30°C. The microcultivation experiments were carried out in 96-well plates with 300 μL of culture medium at 30°C. We used four culture conditions: Synthetic Complete (SC) medium (2% glucose, 0.67% YNB without amino acids and 0.2% complete mix of amino acids); YNB (2% glucose and 0.67% YNB without amino acids); Synthetic Must (SM300) with 300 mg/L of Yeast Assimilable Nitrogen (YAN) (Kessi-Perez et al., 2019a; Molinet et al., 2019); and Synthetic Must (SM60) with 60 mg/L of YAN (Kessi-Perez et al., 2019a; Molinet et al., 2019).

### Genomic Information and Bioinformatic Analyses

The genome sequencing information of ALL, AHG, and BSS strains was obtained from the “1002 yeast genomes project” (Peter et al., 2018). The overlapping DNA contigs of regions A, B, and C were assembled using the *de novo* assembler tool from the Geneious 8.1.8 software (Biomatters, New Zealand). The ORFs inside regions A, B, and C were manually annotated using information from the EC1118 genome and the “1002 yeast genomes project” (Novo et al., 2009; Peter et al., 2018). Then, the ORFs were translated and analyzed using blastp from the blast suit of the NCBI^1^ and SGD databases^2^ for the identification of orthologous genes in the S228c genome (Table 1). The Codon Adaptation Index (CAI) was calculated for each ORF within the three horizontally acquired regions using the CAI calculator (Puigbò et al., 2008). This was performed using the codon usage of *S. cerevisiae* as acceptor species and the codon usage of *Z. bailii* (region B) and *T. delbrueckii* (region A and C) as donor species. We used the codon usage information of *T. delbrueckii* because it was the only one available for a yeast of the *Torulaspora* genus in the Codon Usage Database (Nakamura et al., 2000).

**TABLE 1:** Putative functions and orthologous genes for horizontally acquired genes.

**Order in the HGT region**	***S. cerevisiae* strain**		**EC1118 or S288C**
(annotation name)*	**(donor species)**	**Hypothetical function**	**ortholog**
A1 (0012)	ALL (*Torulaspora sp.*)	Hypothetical protein, putative monoxygenase	No ortholog found
A2 (0023)	ALL (*Torulaspora sp.*)	Enoyl reductase-like, probably dehydrogenase (D-arabinose 1-dehydrogenase)	*YCR102C/YNL134C/YLR460C*
A3 (0034)	ALL (*Torulaspora sp.*)	Sugar (or other) transporter, probably hexose transporter, or plasma membrane high glucose sensor	*RGT2/HXT10/HXT5/STL1/SNF3*
A4 (0045)	ALL (*Torulaspora sp.*)	Aldehyde reductase	*YDR541C/GRE2/ARI1*
A5 (0056)	ALL (*Torulaspora sp.*)	Galactose mutarotase-like, probably UDP-glucose-4-epimerase	*GAL10*
A6 (0078)	ALL (*Torulaspora sp.*)	Fungal transcription factor, probably involved in regulating lysine biosynthesis	*LYS14*
A7 (0089)	ALL (*Torulaspora sp.*)	Arginase	*CAR1*
A8 (0100)	ALL (*Torulaspora sp.*)	Member of the multidrug and toxic compound extrusion (MATE) family, probably Na + -driven multidrug efflux pump	*ERC1/YDR338C*
A9 (0111)	ALL (*Torulaspora sp.*)	Contains a solute binding domain of nucleobase-cation-symport-1 (NCS1) transporter NRT1-like, probably transporter of thiamine or related compound	*THI72/THI7*
A10 (0133)	ALL (*Torulaspora sp.*)	Methyltransferase	*COQ5*
A11 (0144)	ALL (*Torulaspora sp.*)	FAD/FMN-containing dehydrogenase, probably 2-hydroxyglutarate transhydrogenase and minor D-lactate dehydrogenase	*DLD3/DLD2*
A12 (0155)	ALL (*Torulaspora sp.*)	2-Nitropropane dioxygenase or nitronate monooxygenase	*YJR149W*
B24 (0056)	AHG (*Z. bailii*)	Fungal transcription factor	*STB4*
B25 (0012)	AHG (*Z. bailii*)	5-oxoprolinase	*OXP1*
B26 (0023)	AHG (*Z. bailii*)	Member of the Major Facilitator Superfamily (MFS), probably sugar phosphate permease (D-galactonate transporter) or high affinity nicotinic acid plasma membrane permease	*TNA1*
B27 (0034)	AHG (*Z. bailii*)	Flocculation or pseudohypha formation, contains a Flo11 domain	*FLO11*
B28 (0045)	AHG (*Z. bailii*)	Contains a fungal specific transcription factor domain	No ortholog found
C42 (6480)	BSS (*T. microellipsoides*)	Contains a fungal transcription factor domain	*PUT3*
C43 (6491)	BSS (*T. microellipsoides*)	Member of the Major Facilitator Superfamily (MFS), probably allantoate transporter	*SEO1*
C44 (6502)	BSS (*T. microellipsoides*)	Contains a solute binding domain of solute carrier families 5 and 6-like, probably related to amino acid transporter. Oligopeptide transporter.	*FOT1/AVT5/AVT6*
C45 (6524)	BSS (*T. microellipsoides*)	Superoxide dismutase, probably mitochondrial manganese superoxide dismutase	*SOD2*
C46 (6535)	BSS (*T. microellipsoides*)	Ferric reductase with similarity to Fre2p	*FRE7*
C47 (6546)	BSS (*T. microellipsoides*)	Mannitol-1-phosphate/altronate dehydrogenase or mannitol dehydrogenase	*DSF1*
C48 (6557)	BSS (*T. microellipsoides*)	Sugar transporter, probably hexose transporter or transmembrane polyol transporter	*HXT13/HXT17/HXT15/HXT16/GAL2*
C49 (6568)	BSS (*T. microellipsoides*)	Plasma membrane protein, probably ammonium permease	*ATO3*
C50 (6579)	BSS (*T. microellipsoides*)	Galactose mutarotase-like, probably aldose 1-epimerase or UDP-glucose-4-epimerase	*YNR071C/YHR210C/GAL10*
C51 (6612)	BSS (*T. microellipsoides*)	Short-chain dehydrogenase/reductase, probably NADP-dependent mannitol dehydrogenase or peroxisomal 2,4-dienoyl-CoA reductase	*SPS19*
C52 (6623)	BSS (*T. microellipsoides*)	Threonine dehydrogenase or related Zn-dependent dehydrogenase, sorbitol or xylitol dehydrogenase	*XDH1/SOR2/SOR1*
C53 (6634)	BSS (*T. microellipsoides*)	Sugar (or other) transporter, fructose symporter (fructose/H + symporter) or myo-inositol transporter	*FSY1/ITR2/ITR1/CIN10/HXT17/HXT13*
C54 (6645)	BSS (*T. microellipsoides*)	Y’ element-encoded DNA helicase	*YRF1-7/YRF1-6/YRF1-1*
C55 (6656)	BSS (*T. microellipsoides*)	Y’ element-encoded DNA helicase	*YRF1-7/YRF1-6/YRF1-1*

*^∗^ORF order according to the “1002 yeast genomes project” information and annotation name according to the EC1118 strain genome.*

The promoter region of each gene inside regions A, B, and C was scanned for transcription factor binding sites, using the YeTFaSCo database (De Boer and Hughes, 2012). We considered as a promoter the 700 bp upstream of the start codon (ATG) of each ORF and used the whole intergenic region if the distance to the ORF upstream was inferior to 700 bp. We retrieved the information for transcription factor binding sites with non-dubious motifs from YeTFaSCo (Supplementary Table 3).

### Transcriptional Activity Assays

Transcriptional activity was assayed, replacing the target gene with the luciferase reporter, and generating a transcriptional fusion of the promoter region. We replaced the entire coding sequence of the target horizontally acquired genes, from the start codon to the stop codon, using the constructs *Luc-HphMx* or *Luc-NatMx*, which carry the hygromycin (*HphMx*) or nourseothricin (*NatMx*) cassettes as selective markers, respectively (Salinas et al., 2016; Tapia et al., 2018). These genetic constructs encode a destabilized version of the luciferase gene, which contains an ARE sequence for mRNA destabilization and a PEST sequence for proteasome mediated degradation of the protein (Rienzo et al., 2012). The *Luc-HphMx* and *Luc-NatMx* constructs were amplified by PCR using a Phusion flash high-fidelity master mix (Thermo Fisher Scientific, United States) according to the manufacturer’s instructions. The primers used in the PCR reactions contain 20 bp for the amplification of the *Luc-HphMx* or *Luc-NatMx* constructs and 50 bp for direct recombination with the target gene (Supplementary Table 4). The *Luc-HphMx* PCR products were used to transform the ALL and BSS strains, and the *Luc-NatMx* PCR products were used to transform the AHG strain. Strain transformation was carried out using the standard lithium acetate and heat shock protocol (Gietz and Schiestl, 2007). Transformed strains were selected on YPDA plates supplemented with 100 μg/mL hygromycin or 300 μg/mL nourseothricin, as appropriate. Positive colonies were confirmed by standard colony PCR using GoTaq Green Master Mix (Promega, United States) according to the manufacturer’s instructions.

The luciferase expression assays were carried out in microcultivation experiments using 96-well plates with 300 μL of culture medium at 30°C. SC, YNB, SM300, and SM60 culture medias supplemented with 0.1 mM luciferin were used (Salinas et al., 2016). The experiments were conducted in a Synergy HTX plate reader (BioTek, United States) for the simultaneous measurement of OD_600 nm_ and luminescence (Lum) every 30 min. The raw luminescence and OD_600_ data (Supplementary Figure 1) of each strain was normalized, dividing the luminescence by the OD_600_ of the cell culture (Lum/OD_600 nm_). Then, the area under the normalized curves was calculated using the GraphPad Prism 6 software (GraphPad, United States). The total area values were employed as a measurement of the global transcriptional activity for the horizontally acquired genes in a period of 24 h (Supplementary Figure 2; Kessi-Perez et al., 2019b). The *TDH3* ORF was replaced by the luciferase reporter gene in the ALL, AHG, and BSS strains and used as a positive control in the luciferase expression experiments. All assays were carried out with three biological replicas.

### Protein Level Assays

The protein levels were assayed by replacing the stop codon of the target gene by the gene encoding the *mCherry* fluorescent protein, generating a translational fusion. The stop codon was replaced using the *mCherry-HphMx* or *mCherry-NatMx* constructs, which carry either the hygromycin (*HphMx*) or the nourseothricin (*NatMx*) cassettes as selective markers (Tapia et al., 2018). The *mCherry-HphMx* and *mCherry-NatMx* constructs were amplified by PCR using Phusion flash high-fidelity master mix (Thermo Fisher Scientific, United States) according to the manufacturer’s instructions. The primers used in the PCR reactions contain 20 bp for amplification of the *mCherry-HphMx* or *mCherry-NatMx* constructs, and 50 bp for direct recombination with the stop codon of the target genes (Supplementary Table 4). The *mCherry-HphMx* PCR products were used to transform the ALL and BSS strains, and the *mCherry-NatMx* PCR products were used to transform the AHG strain. Strain transformation was carried out using the standard lithium acetate and heat shock protocol (Gietz and Schiestl, 2007). Transformed strains were selected on YPDA plates supplemented with 100 μg/mL hygromycin or 300 μg/mL nourseothricin, as appropriate. Positive colonies were confirmed by standard colony PCR using GoTaq Green Master Mix (Promega, United States), according to the manufacturer’s instructions. Additionally, stop codon removal in the positive colonies was confirmed by Sanger sequencing of the PCR product (Macrogen Inc., South Korea), which was amplified using primers close to the 3‘ end of each horizontally acquired gene (Supplementary Table 4).

Levels of *mCherry* fluorescent protein were assayed in microcultivation experiments using 96-well plates with 300 μL culture medium (SC, YNB, SM300, and SM60) at 30°C. The experiments were conducted in a Synergy HTX plate reader (BioTek, United States), for the simultaneous measurement of OD_600 nm_ and fluorescence (Fluo) every 30 min. Fluorescence data were normalized by the OD_600_ data (Fluo/OD_600 nm_), and the normalized values were corrected by the auto-fluorescence of the wild type strains. Since the *mCherry* fluorescent protein accumulates in the cells over the time course of the experiments, the last time point (24 h) was used to compare the protein levels between horizontally acquired genes. The stop codon of the *TDH3* gene was replaced by the *mCherry* fluorescent protein in strains ALL, AHG, and BSS and used as a positive control in the fluorescence experiments. All assays were performed with three biological replicas.

### Growth Curves and Fermentation Experiments

The growth curves of the strains carrying the luciferase reporter gene (deletion strains) were performed in microcultivation experiments using 96-well plates with 300 μL culture medium (SC, YNB, SM300, or SM60) at 30°C. The OD_600 nm_ of the cell cultures was measured every 30 min using a Synergy HTX plate reader (Biotek, United States). The kinetic parameters were automatically extracted from the growth curves using the Gompertz equation (Zwietering et al., 1990; Yin et al., 2003), which was incorporated as a function in the GraphPad Prism 6 software (GraphPad, United States). The kinetic parameters analyzed for each strain were the latency time (Lag), growth rate (Rate), and growth efficiency (Efficiency) (Warringer and Blomberg, 2003). These phenotypes were normalized, dividing the phenotypes of the strains carrying gene deletions by the phenotypes of their respective wild type strain (ALL, AHG, or BSS), and represented as a heat map using the Heatmapper web server (Babicki et al., 2016). In addition, the phenotypes of each strain were individually compared with respect to the phenotypes of the corresponding wild type version. All experiments were performed with three biological replicas.

The strains carrying the luciferase reporter gene (deletion strains) were also phenotyped by laboratory scale fermentation. These experiments were conducted in 250 mL bottles with 150 mL SM300 medium, using a water bath to control the temperature at 25°C and a magnetic stir plate at 150 rpm to homogenize the cell culture. The fermentations were inoculated with 1 × 10^6^ cells/mL, and progression was monitored every 24 h by CO_2_ release, measured as the weight loss during the time course of the experiment. The fermentations were finalized once stable weight loss measurements were recorded (Kessi-Perez et al., 2019a). The CO_2_ loss curves were fitted to a sigmoid non-linear regression and the first derivative was calculated to obtain the maximal CO_2_ production rate (Vmax) of each strain (Marullo et al., 2006; Molinet et al., 2019). All fermentation experiments were carried out with three biological replicas.

### Statistical Analyses

The statistical comparisons for the different data sets were performed using the Student’s *t*-test, assuming an unpaired data comparison and a non-parametric distribution of the data using the Mann-Whitney test (*p* < 0.05). All statistical analyses were carried out using GraphPad Prism 6 software (GraphPad, United States).

## Results

### Genetic Structure and Putative Biological Function of the Horizontally Acquired Regions

**FIGURE 1:**
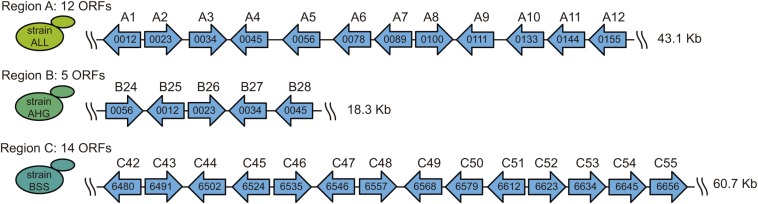
Genetic structure of the horizontally acquired regions. The horizontally acquired regions A, B, and C are present in a single copy per genome in the ALL, AHG, and BSS strains, respectively. The ORFs and their orientation within each region are indicated by arrows. The name inside each ORF corresponds to the annotation according to the genome information of the EC1118 strain. The name above each ORF represents the order in the horizontally acquired region according to the “1002 yeast genomes project” information.

For this study, we used information from the “1002 yeast genomes” initiative. As a means to facilitate the posterior genetic analyses and molecular biology manipulations, we selected three strains with a single copy per genome of the horizontally acquired regions A, B, and C. Thus, we selected the ALL strain for the analysis of region A, the AHG strain for region B, and the BSS strain for region C (Supplementary Table 2). Then, we used the genomic information of the EC1118 strain and of the “1002 yeast genomes project” to assemble a full contig, and subsequently annotate the ORFs (> 100 aa) for each region in the selected strains (Figure 1). In general, the genetic structure of the three regions was analogous to that observed in the EC1118 strain (Novo et al., 2009).

The putative biological function of the ORFs within each region was extracted from the EC1118 strain genome and the “1002 yeast genomes project” information (Table 1). In addition, we translated each ORF *in silico* and performed a blastp alignment against both the NCBI and the fungal SGD databases, allowing us to identify orthologous genes in the reference genome (S288C strain) (Table 1). As expected, most of the genes within the three regions have putative functions in the transport and metabolism of carbon and nitrogen sources (Table 1). Altogether, the observed genetic structure and hypothetical biological functions for the genes encoded in regions A, B, and C in their respective strains (ALL, AHG, and BSS) do not show differences compared to the regions in the EC1118 strains.

### Low Levels of Transcriptional and Translational Activity of the Horizontally Acquired Genes

**FIGURE 2:**
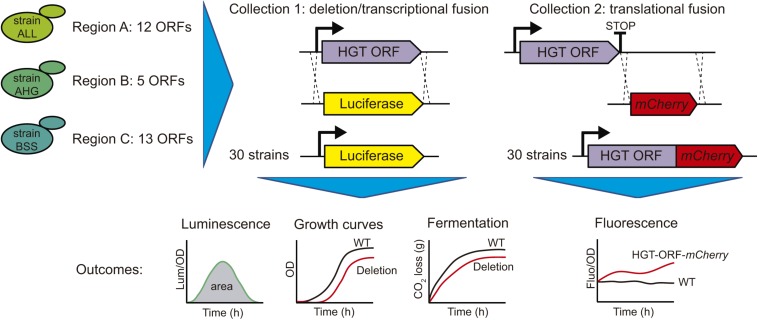
Overview of the molecular characterization of the horizontally acquired regions. In the three selected strains, the ORFs acquired by Horizontal Gene Transfer (HGT) were replaced by the luciferase reporter gene, generating the transcriptional fusion of the promoter region and deletion of the target gene (Collection 1). This collection was assayed for luciferase expression, kinetic parameters using growth curves and fermentation kinetics by laboratory scale fermentations. Similarly, the stop codon of the target genes was replaced by the *mCherry* fluorescent protein, generating a translational fusion (Collection 2). This collection was assayed for *mCherry* fluorescent protein expression.

To assess transcriptional activity and protein levels of each ORF inside regions A, B, and C, we performed a molecular characterization using two different approaches. First, we replaced each ORF with the luciferase reporter gene, generating simultaneously a transcriptional fusion of the promoter region and the deletion of the ORF (Figure 2). This procedure was successfully applied to all the ORFs inside regions A, B, and C, except ORF C54, which could not be successfully transformed and was therefore discarded from posterior analyses. Thus, we generated a collection of 30 strains (collection 1), which was assayed for luciferase expression in four different culture conditions (SC, YNB, SM300, and SM60), allowing us to measure the transcriptional activity as the total area under the normalized luminescence curves (Figure 2). We observed transcriptional activity for eleven horizontally acquired genes in the different culture conditions evaluated, detecting activity in at least one media for ORFs A5, A6, and A10 for region A; B25 and B26 for region B; and C43, C44, C47, C50, C51, and C52 for region C (Supplementary Figures 1, 2). Interestingly, the ORF B25 showed transcriptional activity in the four conditions assayed, whereas ORF B26 was active in SC and fermentative conditions (SM300 and SM60) (Supplementary Figures 1, 2). We also observed transcriptional activity for the *FOT1* gene (C44) in YNB, SM300, and SM60 but not in complete medium (SC) (Supplementary Figures 1, 2), which is consistent with previous reports that show that *FOT1* is expressed under fermentative conditions (Marsit et al., 2015, 2016). Similarly, we were not able to detect transcriptional activity for the *FSY1* gene (C53) in the assayed culture conditions (Supplementary Figure 3), consistent with the described repression of this gene under high levels of glucose (Galeote et al., 2010). Overall, we cannot discard transcriptional activity for the other ORFs present in the analyzed regions, whose promoter regions could have transcriptional activity below the detection level of our destabilized reporter gene. Alternatively, these promoters may have a cryptic behavior or be inactive in the culture conditions assayed. To explore this idea, we scanned the promoter region of each ORF using the YeTFasCo database for yeast transcription factors binding sites, confirming that all ORFs contain multiple and different sites for yeast transcription factor binding in their promoter regions (Supplementary Table 3), suggesting that all the ORFs should be transcriptionally active under specific environmental conditions. Interestingly, most of the ORFs (23 out of 31) contain a *GLN3* motif in their promoter regions, suggesting that they play an important role under nitrogen-limited conditions (Supplementary Table 3).

**FIGURE 3:**
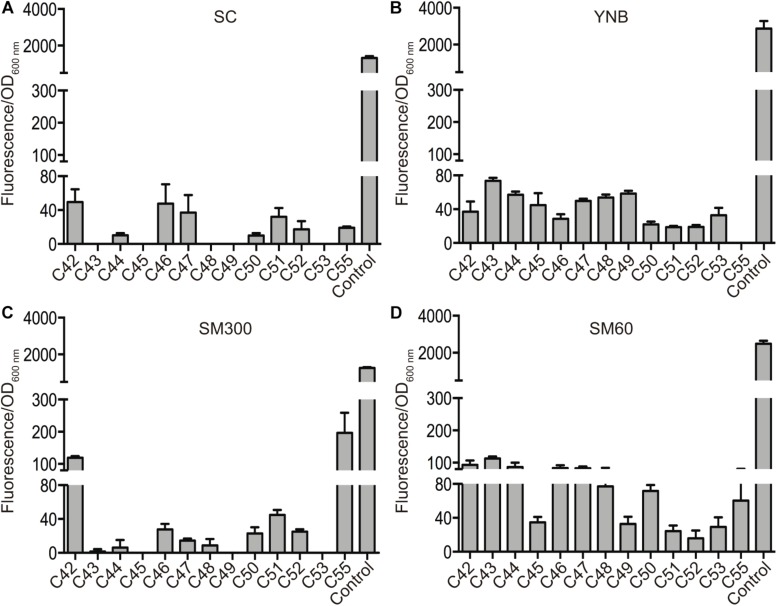
Protein levels of horizontally acquired genes in region C. The protein levels produced by the ORFs inside region C were measured as normalized *mCherry* fluorescence in four culture conditions: SC **(A)**, YNB **(B)**, SM300 **(C)**, and SM60 **(D)**. The translational fusion of the *THD3* gene was used as positive control in all the experiments. The average values of three independent biological replicas (± standard error) are shown.

Secondly, we further characterized regions A, B, and C by assessing the protein level produced by the ORFs inside these regions. For this, we replaced the stop codon of each ORF with the *mCherry* fluorescent protein, generating a translational fusion (Figure 2). This procedure generated a collection of 30 strains (collection 2), which was assayed for *mCherry* fluorescence in the same four different culture conditions (SC, YNB, SM300, and SM60), allowing us to compare the protein levels produced by the horizontally acquired genes (Figure 2). We observed detectable levels of *mCherry* protein in at least one of the culture conditions assayed for all the ORFs analyzed (Supplementary Figure 2). These results support the idea that all the ORFs present in the horizontally acquired regions are indeed active, even though we were not able to detect their transcriptional activity using a destabilized version of the luciferase reporter gene. Alternatively, the ORFs maybe under cryptic transcription, and we were capable of detecting the *mCherry* fusion proteins due to their accumulation over the time course of the experiment (24 h). However, all ORFs that displayed a luciferase signal also produced the *mCherry* fluorescent protein signal (Supplementary Figure 2). Interestingly, the protein levels of six ORFs inside region C (C43, C44, C45, C48, C49, and C53) increased in culture conditions with limited amino acid availability (YNB and SM60) (Figure 3 and Supplementary Figure 4), with the rise being more pronounced in a nitrogen-limited fermentative condition (SM60) (Figure 3 and Supplementary Figure 4). These data confirm the importance of region C for the adaptation of yeast to this environmental condition. Finally, we assessed whether the protein levels measured are influenced by the codon usage differences between the acceptor (*S. cerevisiae*) and donor (*Z. bailii* or *Torulaspora sp.*) species. On calculating the Codon Adaptation Index (CAI) for each ORF of the horizontally acquired regions, we observed a CAI superior to 0.65 for all genes (Supplementary Figure 5), confirming that codon usage does not limit protein synthesis. Altogether, our results support the idea that horizontally acquired genes are active in the strains analyzed, showing low levels of transcriptional and translational activity, but with an increase in protein levels under nitrogen-limited conditions for a subset of ORFs from region C.

### Deletion of Horizontally Acquired Genes Affects Yeast Growth Parameters and Fermentation Kinetics

**FIGURE 4:**
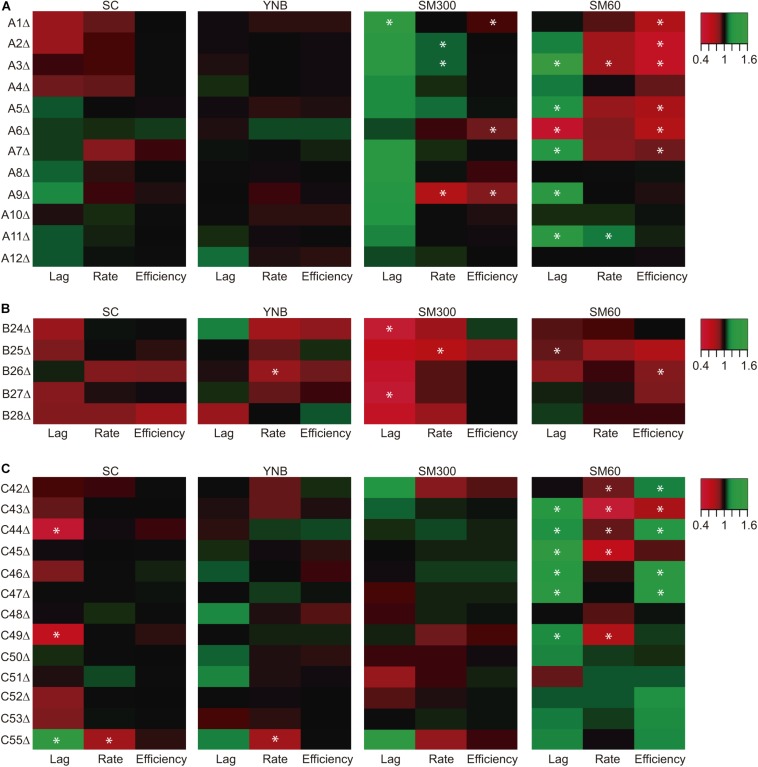
Kinetic parameters for strains carrying deletions in horizontally acquired genes. The strains carrying deletions in different horizontally acquired genes inside region A **(A)**, region B **(B)**, and region C **(C)** were grown in four culture conditions (SC, YNB, SM300, and SM60). The kinetic parameters were extracted from the growth curves for the phenotypic comparison of latency time (Lag), growth rate (Rate) and growth efficiency (Efficiency). The normalized phenotypic values are represented as a heat map where the wild type phenotype has a value equal to 1 in the color scale, and red and green represent lower and higher values of the phenotypes respect to the wild type strain, respectively. Asterisks represent a statistically significant difference between the phenotype of the strain carrying the deletion and its wild type version (*t*-test, *p* < 0.05). The heat map was constructed with the average values of three biological replicas.

Collection 1, whose strains carry the luciferase reporter gene, are also a gene deletion collection for the horizontally acquired genes (Figure 2). Therefore, we performed growth curves for these strains, extracting three kinetic parameters (lag time, growth rate, and growth efficiency), and compared these phenotypes to the respective wild type strains (Figure 4). We also compared the kinetic parameters across different culture conditions using the normalized phenotypic information (Figure 4). In general, we observed that most of the phenotypic differences occur under fermentative conditions (SM300) or nitrogen-limited fermentation conditions (SM60) (Figure 4). Importantly, 20 of the 30 strains carrying single ORF deletions showed a statistically significant phenotypic difference compared to the wild type strain in at least one of the culture conditions assayed, with most of the phenotypic differences observed in SM60 medium (Figure 4). Interestingly, several gene deletions had the combined effects of increasing the lag time and decreasing the growth rate and growth efficiency with respect to the wild type strain (Figure 4). For instance, this finding was observed in ORFs A3 (SM60), A5 (SM60), A7 (SC and SM60), A9 (SM300), C43 (SM60), C44 (SM60), C45 (SM60), and C49 (SM60) (Figure 4), suggesting an important contribution of these genes in the maintenance of yeast kinetic parameters in the assayed conditions.

**FIGURE 5:**
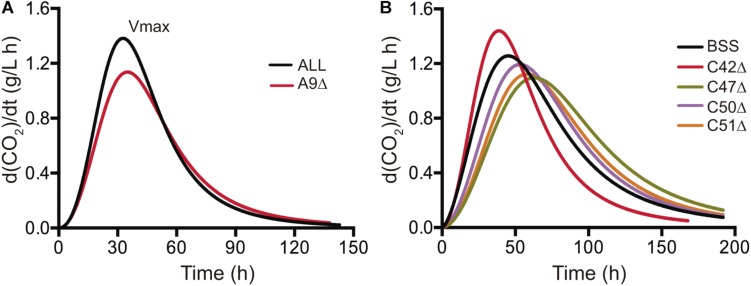
Maximal CO_2_ production rate of strains carrying deletions in horizontally acquired genes. Strains carrying single deletions in different horizontally acquired genes and showing statistically significant differences (*t*-test, *p* < 0.05) with respect to the wild type phenotype are shown for region A **(A)** and region C **(B)**. Small scale fermentations in 150 mL of SM300 medium were performed for each strain and CO_2_ release was measured as weight loss every 24 h. The CO_2_ release curves were fitted to a sigmoid non-linear regression and the first derivative was calculated to obtain the maximal CO_2_ production rate (Vmax, peak of the curves). The average values of three biological replicates are shown.

In order to further investigate the phenotypic effects of our gene deletion collection for horizontally acquired genes over a fermentative phenotype, we performed laboratory scale fermentation in SM300 medium. We monitored the fermentation progress as CO_2_ release, measuring the weight loss every 24 h. Then, we fitted the curves to a sigmoid non-linear regression and calculated the first derivative to extract the maximal CO_2_ production rate (Vmax) (Supplementary Figure 6). This phenotype provided information of when nitrogen is depleted and correlates with the nitrogen demand of the yeast strains (Marullo et al., 2006). The phenotypic information of each strain was compared to the phenotype of the wild type counterpart. Deletion of ORFs A9, C47, C50, and C51 significantly decreased the maximal CO_2_ production rate compared to their respective wild type strains (Figure 5). The results for ORF A9 correlate well with the phenotypes observed in microcultivation experiments, where a significant reduction in the growth rate and growth efficiency was observed in SM300 (Figure 4A). ORF A9 encodes a putative thiamine (vitamin B1) transporter, which is an essential vitamin for amino acid and carbohydrate metabolism during fermentation (Trevelyan and Harrison, 1954). Surprisingly, ORF C42 deletion, encoding a putative transcription factor ortholog to *PUT3*, was the only one that showed a significant increase in the maximal CO_2_ production rate compared to the BSS wild type strain (Figure 5). Altogether, our results provided strong molecular and phenotypic evidence that highlights the importance of horizontally acquired genes in yeast adaptation to the fermentative environment.

## Discussion

The contribution of the HGT process in the configuration of eukaryotic genomes is receiving renewed attention, especially in *S. cerevisiae*, where large-scale sequencing projects have revealed that HGT is a very extensive phenomenon (Legras et al., 2018; Peter et al., 2018). Recently, the molecular incompatibilities between donor and acceptor species have been pointed out, especially in the context of HGT events from bacteria to the yeast clade *Wickerhamiella/Starmerella* (Gonçalves and Gonçalves, 2019; Kominek et al., 2019). The molecular differences between prokaryotes and eukaryotes in the promoter region structure, codon usage, and transcription and translation machineries are barriers to the expression of the horizontally acquired genes (Kominek et al., 2019). If we consider the HGT process in the context of two eukaryotic species (donor and acceptor), the molecular barriers for expression of the horizontally acquired genes should be inferior; however, there is scarce evidence to support this assumption.

We studied the horizontally acquired regions A, B, and C, initially described in the genome of the wine strain EC1118 (Novo et al., 2009), to gain an overview of the molecular processes underlying the horizontally acquired genes inside those regions and in the genetic context of the acceptor species (*S. cerevisiae*). The genes harbored by these regions have been functionally categorized in the transport and metabolism of nitrogen and carbon sources (Novo et al., 2009). In fact, region C contains the *FOT* genes and the *FSY1* gene, which encode for oligopeptide transporters and a fructose transporter, respectively. These genes make a strong contribution to the fermentative capacities of the EC1118 strain (Galeote et al., 2010; Marsit et al., 2015, 2016). However, other genes contained in region C and genes within regions A and B have received less attention, and their involvement in yeast adaptation is less clear. In our work, three strains were chosen that have genetic backgrounds that facilitate the molecular dissection of the horizontally acquired regions, carrying a single copy per genome of these regions, with a similar genetic structure and ORF content to that of the EC1118 strain (Novo et al., 2009). Regions A, B, and C from the selected strains have a similar structure to the regions described in the EC1118 strain (Novo et al., 2009), containing the same number of ORFs inside regions A and B compared to the EC1118 strain (Figure 1). In region C, we identified a lower number of ORFs because we did not consider ORFs encoding proteins of less than 100 amino acids. We also examined the putative function of the annotated ORFs in the selected strains using information from the EC1118 strain, the “1002 yeast genomes project” and a blastp search strategy (Table 1). In general, we confirmed that the annotated ORFs have possible functions in the transport and metabolism of nitrogen and carbon sources (Table 1). Altogether, these results are not unexpected if we consider that selected strains belong to the fermentative Wine/European phylogenetic clade (Supplementary Table 1; Peter et al., 2018).

We assessed the transcriptional and translational activity for each ORF within the horizontally acquired regions A, B, and C (Figure 2). Using the luciferase reporter gene, we observed low levels of transcriptional activity for 11 of the 30 horizontally acquired genes (Supplementary Figures 1, 2). However, the translational reporter (*mCherry*) was consistently detected for all the horizontally acquired genes assayed in at least one culture condition (Supplementary Figure 2). These results allowed us to compare the protein levels produced by the horizontally acquired genes in four culture conditions (Supplementary Figure 2). Interestingly, multiple ORFs within region C suffered an increase in protein levels under nitrogen-limited fermentation conditions (Figure 3 and Supplementary Figure 4), confirming the importance of this genomic region in yeast adaptation to the fermentation process (Marsit et al., 2015, 2016).

The combined results of the transcriptional and translational reporters thus suggest that horizontally acquired genes are transcribed at low levels, which could not be detected using a destabilized reporter gene such as the luciferase construct used in this study (Rienzo et al., 2012). In a similar way, the promoter regions of the horizontally acquired genes may have a cryptic behavior in the *S. cerevisiae* genetic context, generating unstable transcription or mRNAs of rapid degradation (Wyers et al., 2005; Neil et al., 2009). This hypothesis is also supported by the consistent signal obtained using the *mCherry* fluorescent protein, which is a stable translational reporter that accumulates during the time course of the experiments (Deluna et al., 2010). Importantly, regions A and C are subtelomeric according to the EC1118 strain genome (Novo et al., 2009). Therefore, the ORFs in both these regions could suffer potential subtelomeric repression and be activated only under specific environmental conditions (Ai et al., 2002). A simple and alternative explanation for the low levels of transcriptional activity observed in the horizontally acquired genes is the limited number of environmental conditions used in this work, where only four culture conditions were assayed. Consequently, we cannot discard transcriptional activity for the analyzed ORFs in other culture conditions, especially considering the multiple yeast transcription factor binding sites present in their promoter regions (Supplementary Table 3). Furthermore, we also confirmed through the CAI calculation for each ORF that codon usage is not a bias for protein synthesis in the context of *S. cerevisiae* (Supplementary Figure 5), observing an average CAI higher than 0.65 for the horizontally acquired genes, which is also higher than the average CAI for the *S. cerevisiae* genome (Ghaemmaghami et al., 2003; Jansen et al., 2003).

We assayed the growth kinetic parameters and the maximal CO_2_ production rate in a gene deletion collection of strains (Figure 2) and compared these phenotypes to the wild type strains. We found that the single deletion of 20 ORFs produced a significant modification of the kinetic parameters in at least one of the assayed culture conditions, obtaining most of the phenotypic changes in SM60 (Figure 4). Importantly, SM60 is a fermentation medium that mimics wine fermentation conditions but contains a limited amount of nitrogen, generating a stressful environment for yeast growth (Kessi-Perez et al., 2019a; Molinet et al., 2019). In a similar way, the single deletion of five ORFs significantly changed the maximal CO_2_ production rate (Figure 5). Interestingly, we observed a consistent result between growth parameters and maximal CO_2_ production rate for ORF A9 deletion, increasing the lag time and decreasing the growth rate, growth efficiency, and CO_2_ production rate (Figures 4, 5). This ORF (A9) encodes for a putative thiamine (vitamin B1) transporter, which is a co-factor for many enzymes that participate in the metabolism of amino acids and carbohydrates (Jurgenson et al., 2009). In the yeast fermentation context, thiamine decreases the level of pyruvate and increases the amount of carboxylase enzymes, generating an increase in the fermentation rate (Trevelyan and Harrison, 1954). This is consistent with our results, where ORF A9 deletion decreased the maximal CO_2_ production rate (Figure 5).

In conclusion, our results confirm the importance of the deletion approach for yeast functional genomics (Giaever and Nislow, 2014) and provide molecular evidence for the transcriptional and translational activity of the horizontally acquired genes and their importance for niche-specific adaptation, particularly in the fermentative environment. Further complementation of these results with the generation of an overexpression collection of these genes will allow us to uncover novel cellular functions for this important set of genes (Stevenson et al., 2001; Sopko et al., 2006).

## Data Availability Statement

The datasets generated for this study are available upon request to the corresponding author.

## Author Contributions

FS designed the research and wrote the manuscript with insight from all the authors. JD, CB, EK-P, CV, and MD performed the bioinformatics analyses, laboratory experiments, and analyzed the data. FC, GL, CM, and FS supervised the work and provided the reagents and financial support.

## Conflict of Interest

The authors declare that the research was conducted in the absence of any commercial or financial relationships that could be construed as a potential conflict of interest.

## Footnotes

1 https://blast.ncbi.nlm.nih.gov/Blast.cgi

2 https://yeastgenome.org/blast-sgd
