# Supplementary material for: Transcriptional Activity and Protein Levels of Horizontally Acquired Genes in Yeast Reveal Hallmarks of Adaptation to Fermentative Environments
Source: Front Genet. 2020 Apr 30;11:293. doi: 10.3389/fgene.2020.00293 (PMC7212421; doi:10.3389/fgene.2020.00293)
Supplement: Supplementary file 1 [file Data_Sheet_1.PDF]

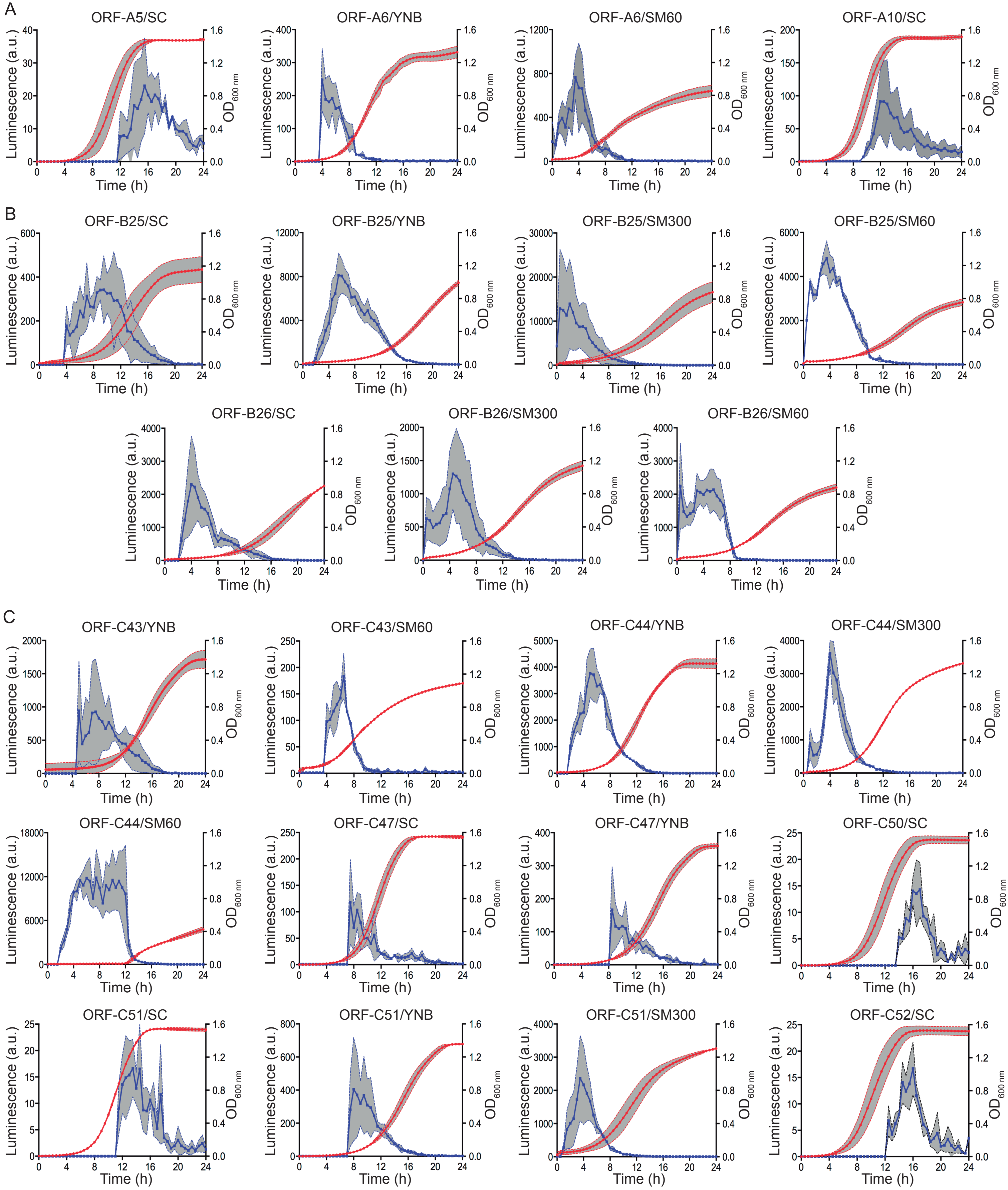

Supplementary Figure 1. Raw data of luciferase expression and growth curves used in the transcriptional activity analysis. The luciferase expression was measured as luminescence (blue curves) and the growth curve was recorded as OD600nm (red curves) for each yeast culture. The horizontally acquired genes inside regions A (panel A), B (panel B), and C (panel C) that showed a positive luciferase signal are shown in the corresponding culture condition. The plots show the average of three biological replicas with the standard deviation represented as shadow regions.
