# Supplementary material for: Transcriptional Activity and Protein Levels of Horizontally Acquired Genes in Yeast Reveal Hallmarks of Adaptation to Fermentative Environments
Source: Front Genet. 2020 Apr 30;11:293. doi: 10.3389/fgene.2020.00293 (PMC7212421; doi:10.3389/fgene.2020.00293)
Supplement: Supplementary file 2 [file Data_Sheet_2.PDF]

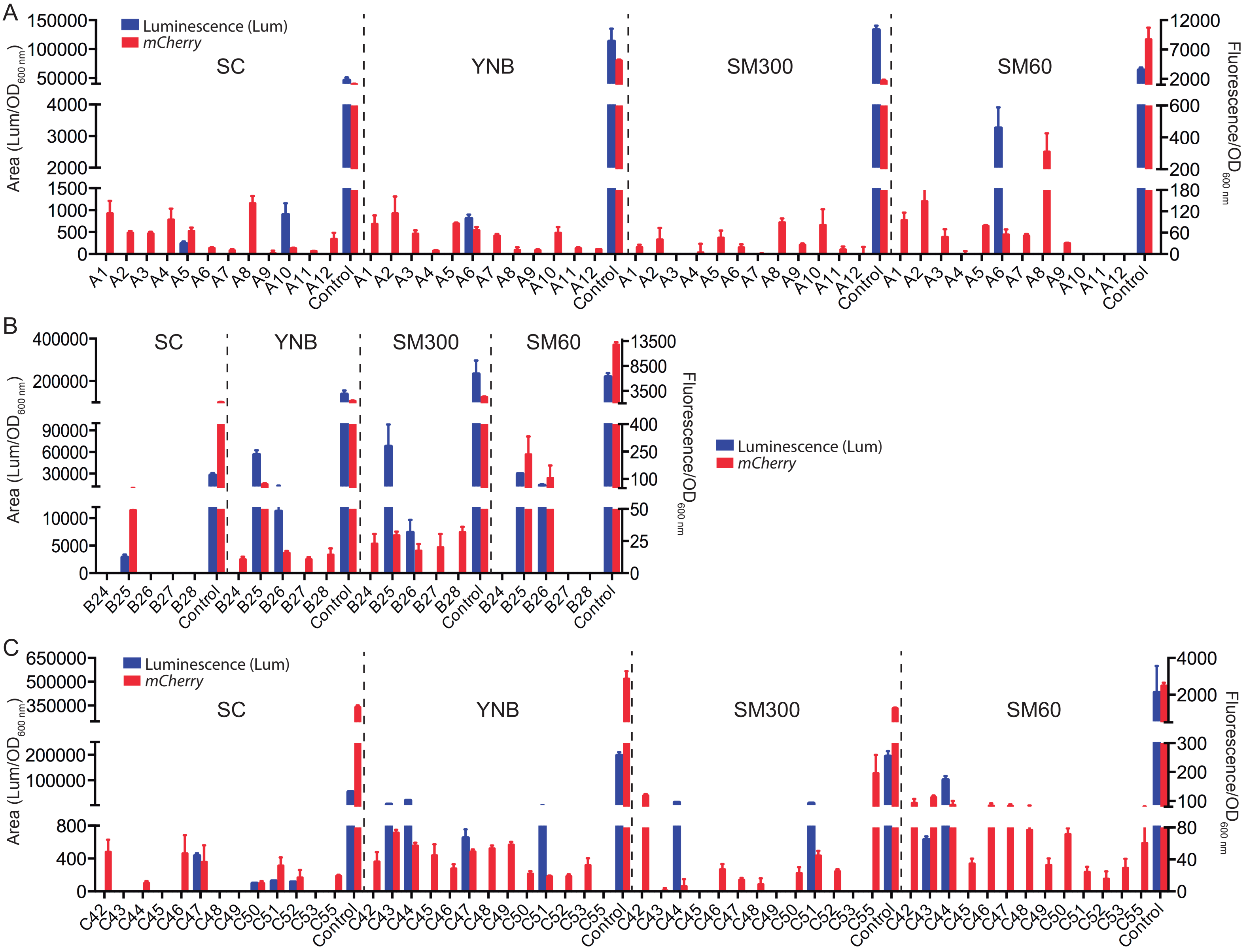

Supplementary Figure 2. Transcriptional and translational activity for horizontally acquired genes. The transcriptional and translational activity for each ORFs inside region A (panel A), region B (panel B) and region C (panel C) was assayed using the luciferase reporter gene and the mCherry fluorescent protein, respectively. The transcriptional activity is shown as the area under the normalized luminescence curves (Lum/OD) during a period of 24 hours. The translational activity correspond to the final point (24 hours) of normalized mCherry fluorescence (Fluo/OD). The strains were assayed in four culture conditions: SC, YNB, SM300 and SM60. The transcriptional and translational fusions for *THD3* gene were used as positive control in all the experiments. The average of three biological replicas with its standard error is shown.
