# Supplementary material for: Transcriptional Activity and Protein Levels of Horizontally Acquired Genes in Yeast Reveal Hallmarks of Adaptation to Fermentative Environments
Source: Front Genet. 2020 Apr 30;11:293. doi: 10.3389/fgene.2020.00293 (PMC7212421; doi:10.3389/fgene.2020.00293)
Supplement: Supplementary file 3 [file Data_Sheet_3.PDF]

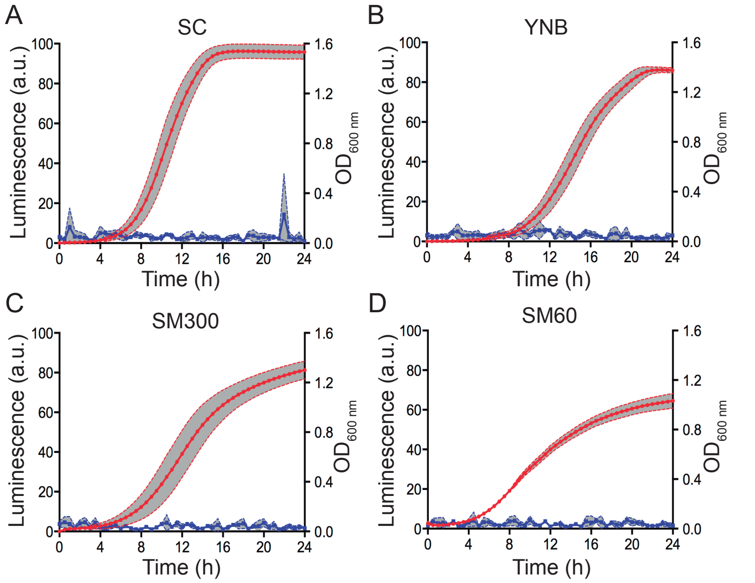


**Supplementary Figure 3. Raw data of luciferase expression and growth curves for the ORF-C53**. Background levels of luciferase expression were detected for the ORF-C53 (*FSY1* gene), measured as luminescence (blue curves), whereas the growth curves were recorded as OD600nm (red curves). The panels A, B, C and D shows the levels of luciferase expression and growth curves in SC, YNB, SM300 and SM60 culture mediums, respectively. The plots show the average of three biological replicas with the standard deviation represented as shadow regions.
