# Supplementary material for: Transcriptional Activity and Protein Levels of Horizontally Acquired Genes in Yeast Reveal Hallmarks of Adaptation to Fermentative Environments
Source: Front Genet. 2020 Apr 30;11:293. doi: 10.3389/fgene.2020.00293 (PMC7212421; doi:10.3389/fgene.2020.00293)
Supplement: Supplementary file 4 [file Data_Sheet_4.PDF]

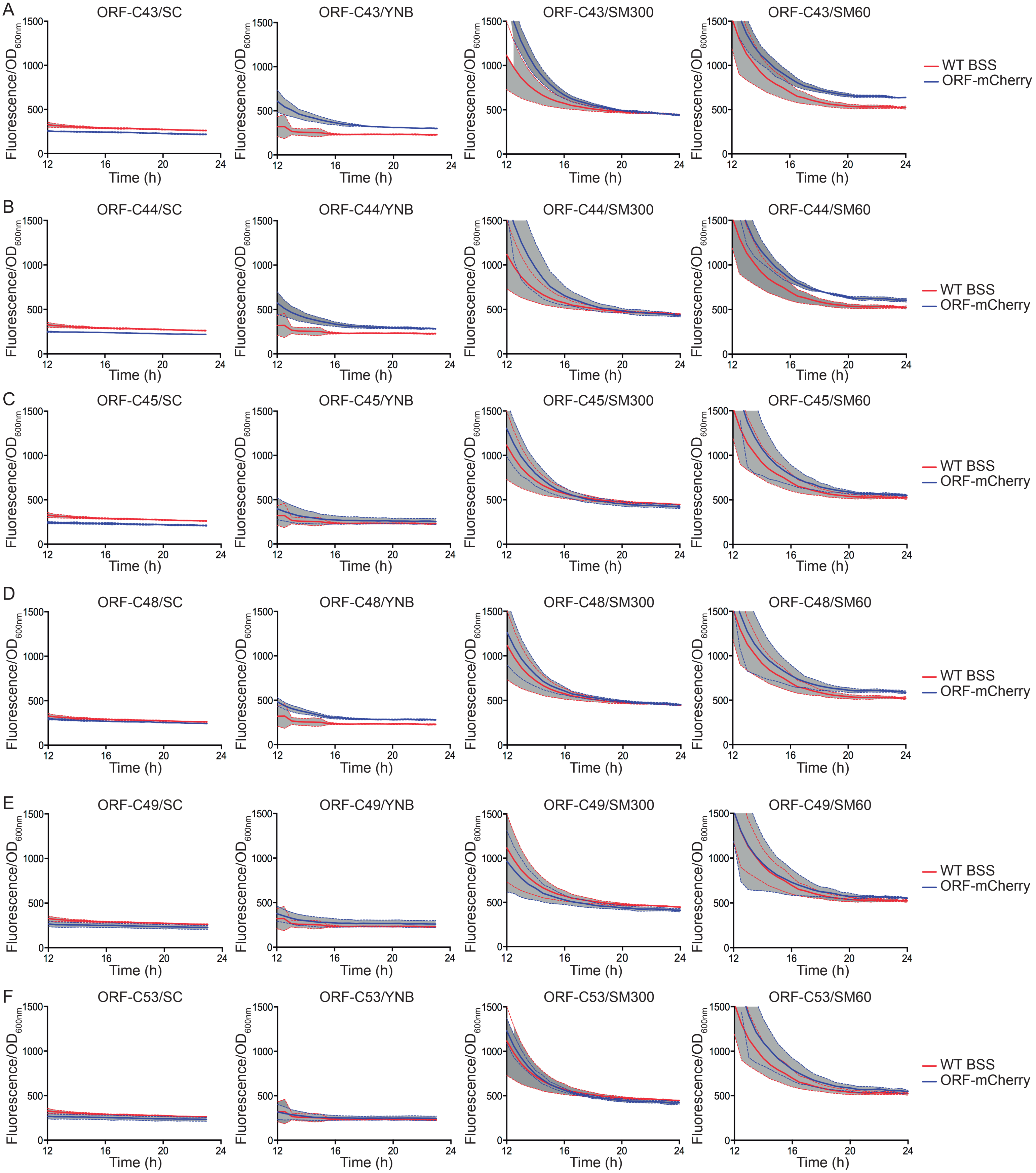

Supplementary Figure 4. Normalized *mCherry* protein levels for a subset of ORFs inside region C. A subset of six ORFs from region C showed an increased in *mCherry* protein levels in YNB and SM60 respect to Sc and SM300 culture mediums. The *mCherry* protein levels for the ORFs C43, C44, C45, C48, C49 and C53 are shown in four culture conditions in panels A, B, C, D, E and F, respectively. The plots show the average of three biological replicas with the standard deviation represented as shadow regions.
