# Supplementary material for: Transcriptional Activity and Protein Levels of Horizontally Acquired Genes in Yeast Reveal Hallmarks of Adaptation to Fermentative Environments
Source: Front Genet. 2020 Apr 30;11:293. doi: 10.3389/fgene.2020.00293 (PMC7212421; doi:10.3389/fgene.2020.00293)
Supplement: Supplementary file 5 [file Data_Sheet_5.PDF]

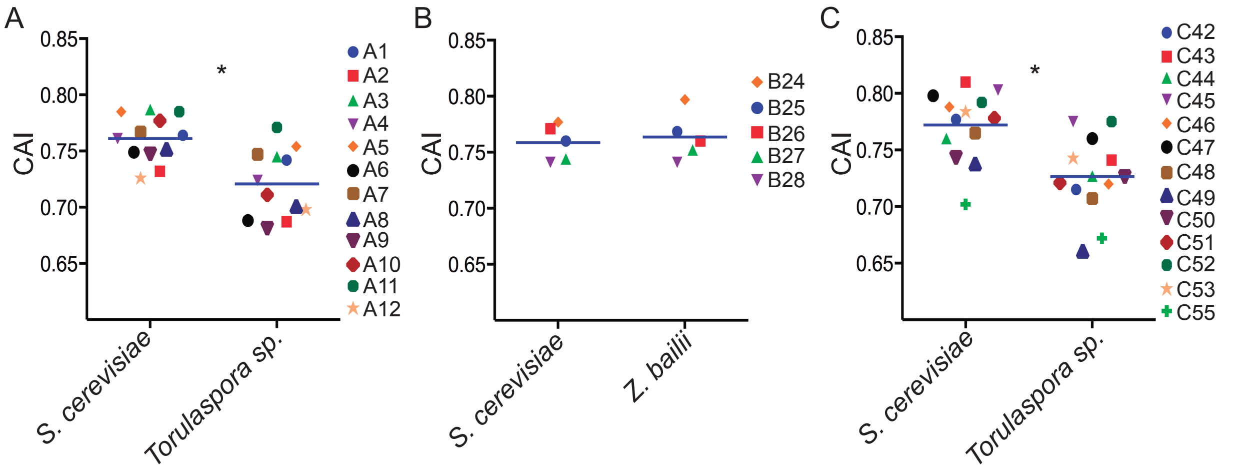

Supplementary Figure 5. Codon adaptation index for the horizontally acquired genes. The Codon Adaptation Index (CAI) was calculated for each ORF inside region A (panel A), region B (panel B) and region C (panel C), utilizing the codon usage of the acceptor (*S. cerevisiae*) and donor (*Z. bailii* and *Torulaspora sp.*) species. The asterisks represent a statistically significant difference between the average CAI for the ORFs in the acceptor and donor species (t-test, $p < 0.05$).
