# Supplementary material for: Transcriptional Activity and Protein Levels of Horizontally Acquired Genes in Yeast Reveal Hallmarks of Adaptation to Fermentative Environments
Source: Front Genet. 2020 Apr 30;11:293. doi: 10.3389/fgene.2020.00293 (PMC7212421; doi:10.3389/fgene.2020.00293)
Supplement: Supplementary file 6 [file Data_Sheet_6.PDF]

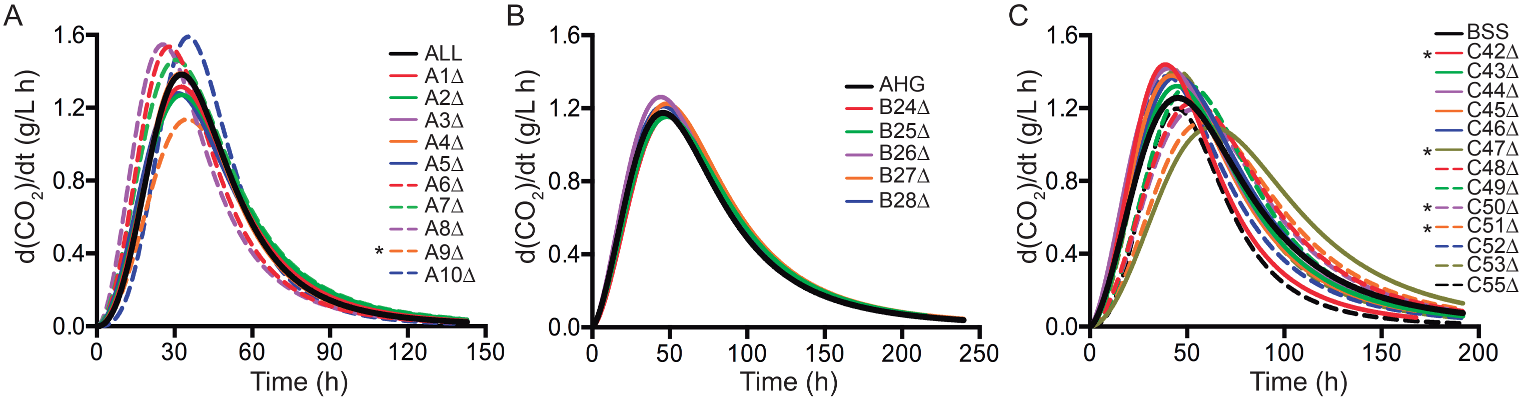

Supplementary Figure 6. Maximal CO_2 production rate for strains carrying deletions in horizontally acquired genes. Small scale fermentations in 150 mL of SM300 medium were performed for the strains carrying deletions in different horizontally acquired genes inside regions A (panel A), B (panel B) and C (panel C). The CO_2 release curves were fitted to a sigmoid non-linear regression and the first derivative was calculated to obtain the maximal CO_2 production rate (Vmax, peak of the curves). The average of three biological replicas is shown. The asterisks in the legend represent a statistically significant difference between the phenotype (Vmax) of the strain carrying the deletion and its wild type version (t-test, $p < 0.05$).
